# Behavioral Responses of the Invasive *Halyomorpha halys* (Stål) to Traps Baited with Stereoisomeric Mixtures of 10,11-Epoxy-1-bisabolen-3-OL

**DOI:** 10.1007/s10886-015-0566-x

**Published:** 2015-04-09

**Authors:** Tracy C. Leskey, Ashot Khrimian, Donald C. Weber, Jeffrey C. Aldrich, Brent D. Short, Doo-Hyung Lee, William R. Morrison

**Affiliations:** 1 USDA-ARS, Appalachian Fruit Research Station, Kearneysville, WV USA; 2 USDA-ARS Invasive Insect Biocontrol & Behavior Laboratory, Beltsville, MD USA; 3 Department of Life Science, Gachon University, Seongnam-si, Kyeonggi-do, South Korea

**Keywords:** Semiochemicals, Behavior, *Halyomorpha halys*, Monitoring, Integrated pest management, Aggregation pheromones, Hemiptera, Pentatomidae, Invasive pest

## Abstract

The brown marmorated stink bug, *Halyomorpha halys*, is an invasive insect in the United States that is capable of inflicting significant yield losses for fruit, vegetable, and soybean growers. Recently, a male-produced aggregation pheromone of *H. halys* was identified as a 3.5:1 mixture of (3*S*,6*S*,7*R*,10*S*)-10,11-epoxy-1-bisabolen-3-ol and (3*R*,6*S*,7*R*,10*S*)-10,11-epoxy-1-bisabolen-3-ol, two stereoisomers of a natural sesquiterpene with a bisabolane skeleton, potentially existing in 16 stereoisomeric forms. In this study, we assessed attraction to pheromonal and non-pheromonal stereoisomeric mixtures of 10,11-epoxy-1-bisabolen-3-ol, which are easier to synthesize than single isomers, and evaluated dose-dependent responses to attractive mixtures in field trials. Some treatments not containing the natural pheromone components were moderately active in field-trapping studies, signifying that some stereoisomers of 10,11-epoxy-1-bisabolen-3-ol are sufficiently similar to the true pheromone in structure to trigger behavioral responses. Importantly, we found that mixtures of stereoisomers containing pheromone components were also highly attractive to *H. halys*, even in the presence of multiple “unnatural” stereoisomers. Further, adult and nymphal captures were dose-dependent, regardless of whether the lure contained pheromonal or non-pheromonal components. Our findings of attraction to pheromonal and non-pheromonal stereoisomers and lack of inhibition from non-pheromonal stereoisomers of 10,11-epoxy-1-bisabolen-3-ol increase the flexibility of developing pheromone-based products for *H. halys*.

**Electronic supplementary material:**

The online version of this article (doi:10.1007/s10886-015-0566-x) contains supplementary material, which is available to authorized users.

## Introduction

*Halyomorpha halys* (Stål), the brown marmorated stink bug (BMSB), is an invasive insect native to China, Taiwan, Korea, and Japan, which was accidently introduced into the United States sometime in the mid–late 1990s (Hoebeke and Carter 2003). Currently, as of June 2014 (www.stopbmsb.org for updates), *H. halys* is well established throughout the mid-Atlantic region (Leskey et al. 2012c), and has been officially detected in 43 states and the District of Columbia. *Halyomorpha halys* is a polyphagous pest of many crops in Asia (Lee et al. 2013a; Panizzi et al. 2000), North America, and Europe (Leskey et al. 2012a; Rice et al. 2014). In 2010, *H. halys* populations increased dramatically and attacked many crops in the mid-Atlantic region. Damage in commercial orchard crops reached critical levels with some growers experiencing serious losses in stone and pome fruit (Leskey et al. 2012a).

Native stink bugs have long been managed with broad-spectrum insecticides, but since the passage of the Food Quality Protection Act in 1996, many broad-spectrum materials have been banned or severely curtailed through regulatory measures. This has caused populations of native stink bugs, long considered to be secondary pests in certain crops, to become more prevalent. Subsequently, as *H. halys* has become well established, populations have exerted tremendous season-long pest pressure, complicating the IPM programs for many crops. A number of broad spectrum insecticides have been identified as being effective against *H. halys* (Lee et al. 2013b; Leskey et al. 2012b; Nielsen et al. 2008); however, their use leads to profound changes in management and severe disruption to previously established IPM programs (Leskey et al. 2012a).

Native stink bug species typically have been monitored in cropping systems using sweep nets, beating samples, pheromone-baited traps, and/or blacklight traps (Borges et al. 2011; Kamminga et al. 2009; Krupke et al. 2001; Leskey and Hogmire 2005). For *H. halys*, pyramid traps baited with the aggregation pheromone [methyl (2*E*, 4*E*, 6*Z*)-decatrienoate] of *Plautia stali* Scott have been used to monitor populations (Joseph et al. 2013; Leskey et al. 2012d; Nielsen et al. 2011), because this compound was found to serve as a kairomone for *H. halys* (Aldrich et al. 2007; Khrimian et al. 2008). Although *H. halys* are attracted to methyl (2*E*, 4*E*, 6*Z*)-decatrienoate late in the season, adults do not respond to this chemical earlier, throughout much of the growing season (Leskey et al. 2012d), making it difficult to quantify the abundance and distribution of pest populations. Furthermore, some commercial lure formulations of this kairomone have had problems with release rates, contributing to low captures (Joseph et al. 2013). Black light traps also have been used to track the relative seasonal abundance (Nielsen et al. 2011), to monitor spread (Nielsen et al. 2013) and to identify landscape-level risk factors (Rice et al. 2014; Wallner et al. 2014). However, light traps are less specific and practical for growers to use to monitor populations on farms due to cost and labor.

Until recently, the pheromone of *H. halys* had not been identified, thereby limiting options for season-long monitoring at farm-scape levels. The two-component male-produced aggregation pheromone, consisting of (3*S*,6*S*,7*R*,10*S*)-10,11-epoxy-1-bisabolen-3-ol and (3*R*,6*S*,7*R*,10*S*)-10,11-epoxy-1-bisabolen-3-ol, of *H. halys* (Khrimian et al. 2014a) is attractive to males, females, and to nymphs and is synergized by the addition of methyl (2*E*, 4*E*, 6*Z*)-decatrienoate (Weber et al. 2014a). While the two natural pheromone components are difficult to synthesize in pure form, they are relatively easy to produce as mixtures of stereoisomers. Thus, to develop and optimize commercially viable formulations of the pheromone, it is important to understand how other non-pheromonal stereoisomers of 10,11-epoxy-1-bisabolen-3-ol may affect attractiveness to *H. halys*, since they are byproducts of chemical syntheses. Therefore, the goal of this study was to assess field responses of *H. halys* to a range of stereoisomers of 10,11-epoxy-1-bisabolen-3-ol, specifically to: 1) evaluate behavioral responses to pheromonal and non-pheromonal stereoisomers of *H. halys*; 2) determine if stereoisomeric mixtures containing pheromone and non-pheromone components are as attractive as mixtures containing only pheromone components; and 3) establish dose-dependent responses to pheromonal and non-pheromonal stereoisomers.

## Methods and Materials

### Chromatography Used in Synthetic Chemistry

Gas chromatography (GC) analyses were performed on an Agilent Technologies 6890 N instrument equipped with a flame ionization detector and a DB-5 capillary column (30 m × 0.32 mm i.d. × 0.25 μm film). Hydrogen was used as carrier gas at 1 ml min^−1^. Column temperature was maintained at 50 °C for 3 min., and then raised to 270 °C at 10 °C min^−1^. Electron impact ionization (EI) mass spectra were obtained at 70 eV with an Agilent Technologies 5973 mass selective detector (MS) interfaced with a 6890 N GC system equipped with either a 30 m × 0.25 mm i.d. × 0.25 μm film HP-5MS column, or one of the chiral columns described previously (Khrimian et al. 2014a). The HP-5MS column temperature was maintained at 50 °C for 5 min, and then raised to 270 °C at 10 °C min^−1^. Helium was used as a carrier gas at 1 ml min^−1^. Thin layer chromatography (TLC) analyses were conducted on Whatman AL SIL G/UV plates using a 20 % ethanol solution of phosphomolybdic acid, and/or UV for visualization of compounds. Flash chromatography was carried out with 230–400 mesh silica gel (Fisher Scientific, Fair Lawn, NJ, USA).

### Lure Preparation

All reagents and solvents were purchased from Aldrich Chemical Company (Milwaukee, WI, USA) unless otherwise specified. While (*S*)-(−)-citronellal (97 % ee) was obtained from the supplier above, (*R*)-(+)-citronellal (98 % ee) was purchased from Takasago International (Tokyo, Japan). (3*R*,6*R*,7*S*,10*S*)-10,11-Epoxy-1-bisabolen-3-ol (RRSS, lure #1, Table 1), (3*R*,6*R*,7*R*,10*R*)-10,11-epoxy-1-bisabolen-3-ol (RRRR, part of lure #7), and (3*R*,6*R*,7*R*,10*S*)-10,11-epoxy-1-bisabolen-3-ol (RRRS, part of lure #7) were described in Khrimian et al. (2014a). (3*S*,6*S*,7*S*,10*S*)-10,11-Epoxy-1-bisabolen-3-ol (SSSS, part of lure #4) and (3*S*,6*S*,7*S*,10*R*)-10,11-epoxy-1-bisabolen-3-ol (SSSR, part of lure #4) were described in Khrimian et al. (2014b).

**Table 1:** 10,11-Epoxy-1-bisabolen-3-ol stereoisomers and mixtures tested in 2011–2013 in field trials against *Halyomorpha halys* in West Virginia and Maryland

Lure	Lure description^a^	Loading (mg)^ab^	*H. halys* pheromone present (mg)
#1	RRSS	2	No
#2	*cis*-(7*S*)-10,11-Epoxy-1-bisabolen-3-ols (four stereoisomers)	8	No
#3	#2 plus *trans*-(7*S*)-10,11-Epoxy-1-bisabolen-3-ols, 3:1 (total eight stereoisomers)	8 + 2.7	No
#4	RRSS plus 1:1 mixture of SSSS and SSSR	8 + 0.3	No
#5	#3 plus mixture A^a^	8 + 0.8	No
#6	*cis*-(7*R*)-10,11-Epoxy-1-bisabolen-3-ols plus *trans*-(7*R*)-10,11-epoxy-1-bisabolen-3-ols, 3:1	8 + 2.7	SSRS (2.0)^c^, RSRS (0.8)^d^
#7	RRRR plus RRRS	2 + 2	No
#8	*cis*-(7*R*)-10,11-Epoxy-1-bisabolen-3-ols (four stereoisomers)	8	SSRS (2.0)
#9	*trans*-(7*R*)-10,11-Epoxy-1-bisabolen-3-ols (four stereoisomers)	8	RSRS (2.5)
#10	#6 plus methyl (2*E*, 4*E*, 6*Z*)-decatrienoate^e^	10.7 + 119/66^f^	SSRS (2.0), RSRS (0.8)
#11	Crude mixture of *cis*-(7*R*)-10,11-Epoxy-1-bisabolen-3-ols and *trans*-(7*R*)-10,11-epoxy-1-bisabolen-3-ols in 1:1.6 ratio	24	SSRS, RSRS
#12	Mimics #6 but uses only one chromatographic purification step instead of two	10.7	SSRS (2.0), RSRS (0.8)
#13	Crude 10,11-Epoxy-1-bisabolen-3-ol (all 16 stereoisomers); one chromatographic purification throughout whole synthesis. Ratio of *cis*/*trans* 1:1.5–2.0	44	SSRS, RSRS
#14	Crude 10,11-Epoxy-1-bisabolen-3-ol (all 16 stereoisomers); no chromatographic purification throughout whole synthesis. Ratio of *cis*/*trans* 1:1.5–2.0	46	SSRS, RSRS

^a^See Material and Methods and Supplementary Material

^b^Impregnated grey rubber septa. West Pharmaceutical Services, Inc., Exton, PA

^c^SSRS content was determined by gas chromatographic (GC) analysis on Chiraldex G-TA column (Khrimian et al. 2014a), and is the major *H. halys* pheromone component

^d^RSRS content was determined by GC analysis on a Hydrodex β-6TBDM column (Khrimian et al. 2014a), and is the minor *H. halys* pheromone component

^e^Polyethylene sachets from Sterling (2011–2012) and AgBio (2013)

^f^AgBio MDT lures contained 66 mg whereas Sterling MDT lures contained 119 mg of MDT.

Lures prepared for field deployment included those aimed at establishing the responses to pheromonal and non-pheromonal stereoisomers of 10,11-epoxy-1-bisabolen-3-ol. Details associated with each treatment, including components and loadings, can be found in Table 1. Specific synthetic pathway details for chemical mixtures that make up treatments referred to as #2, #3, #5, #6, #8, #9, #11, #12, #13, and #14 are given below.

### Syntheses of cis- and trans-(7R)-10,11-epoxy-1-bisabolen-3-ols (Fig. 1)

**Fig. 1:**
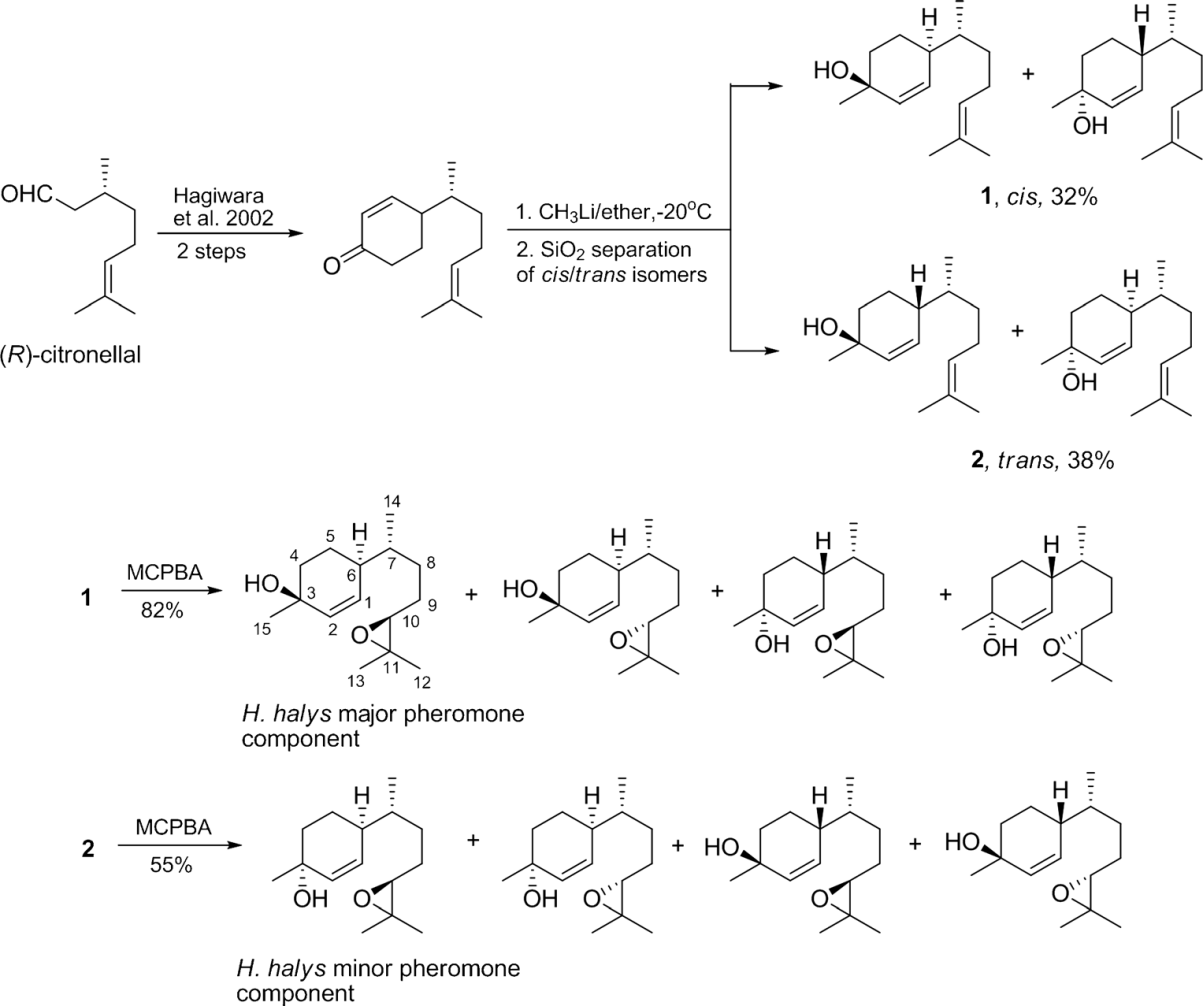
Syntheses of stereoisomeric mixtures of *cis*-(7*R*)-10,11-epoxy-1-bisabolen-3-ol (Lure **#**8), containing *Halyomorpha halys* major pheromone component (3*S*, 6*S*, 7*R*, 10*S*)-10,11-epoxy-1-bisabolen-3-ol (SSRS), and *trans*-(7*R*)-10,11-epoxy-1-bisabolen-3-ol (Lure **#**9), containing *H. halys* minor pheromone component (3*R*, 6*S*, 7*R*, 10*S*)-10,11-epoxy-1-bisabolen-3-ol (RSRS)

(*R*)-Citronellal was converted to (7*R*)-4-(6-methylhept-5-en-2-yl) cyclohex-2-enone (Hagiwara et al. 2002), and the latter reacted with methyl lithium as described in Zahn et al. (2008) with some modifications. A dry three-neck round-bottom flask, fitted with a dropping funnel, magnetic stirrer, thermometer, and N_2_ inlet, was charged with (7*R*)-4-(6-methylhept-5-en-2-yl) cyclohex-2-enone (3.006 g, 14.57 mmol) and 90 ml of dry ether. The flask was cooled to −20 °C and methyl lithium (12 ml of 1.6 M in ether; 19.2 mmol) was added slowly to the solution while maintaining the reaction temperature between −15° and −20 °C. After addition (about 30 min.), the reaction mixture was allowed to warm to room temperature within 2 hr and stirred for another 2 hr or until TLC showed very little starting ketone present. The reaction mixture was cooled to 0° to −5 °C, then treated with a saturated ammonium chloride solution until pH reached ~7, and the solvent layers were separated. The aqueous layer was extracted with hexane:ether, 1:1; the combined organic extracts were washed with brine and dried with sodium sulfate. After evaporation of the solvent, the crude mixture was subjected to flash chromatography on silica gel using hexane:ethyl acetate, 8:1 to 5:1, respectively. A fraction (1.02 g, 32 %) with Rf 0.25 (hexane:ethyl acetate, 5:1) was identified as a diastereomeric mixture, **1**, of two *cis*-(7*R*)-1,10-bisaboladien-3-ols, and a more polar fraction (1.25 g, 38 %) with Rf 0.17 (hexane/ethyl acetate, 5:1) was found to be a mixture, **2**, of two *trans*-(7*R*)-1,10-bisaboladien-3-ols. The *cis-* and *trans*-Bisaboladienols were well-separated on an HP-5MS capillary column during GC/MS analysis. However, two *cis*-stereoisomers and two *trans-*stereoisomers were not separated from each other. For assignment of relative configurations (*cis/trans*) see Khrimian et al. (2014a). Mass spectra of *cis-* and *trans*-Bisaboladienols were identical with those previously published (Zahn et al. 2008).

A mixture of *cis*-Bisaboladienols (**1**, two diastereomers, 148 mg, 0.67 mmol) was stirred with *meta*-chloroperbenzoic acid (MCPBA, 159 mg of 80–85 % pure, 0.74 mmol) in the presence of anhydrous sodium acetate (61 mg) in dichloromethane (DCM, 4 ml, dried over CaH_2_) at 0–5 °C for 3.5 hr. Water (5 ml) was added, and the layers were separated. The aqueous layer was extracted with dicholoromethane (3 × 5 ml), and the combined organic extracts washed with a sodium bicarbonate solution to remove *m*-chlorobenzoic acid that had formed, washed with brine, and then dried with sodium sulfate. After evaporation of the solvent, the residue was flash-chromatographed on silica (hexane:ethyl acetate, 2:1) to give the *cis*-(7*R*)-10,11-epoxy-1-bisabolen-3-ols (four stereoisomers, 131 mg, 82 %), Rf 0.25 (hexane/ethyl acetate, 2:1). GC/EI-MS (*m/z*, relative abundance): 220 (2, M^+^-18), 165 (26), 138 (30), 134 (50), 132 (39), 119 (43), 109 (40), 105 (31), 93 (72), 91 (50), 79 (45), 77 (36), 71 (59), 69 (26), 67 (27), 59 (29), 55 (33), 43 (100), 41 (42). The data are in agreement with those previously published (Zahn et al. 2008) and matched those for the main male-specific compound (SSRS, Fig. 1) found in *H. halys* extract (Khrimian et al. 2014a). This mixture was tested in the field as lure **#**8 (Fig. 1, Table 1).

A mixture of *trans*-bisaboladienols (**2**, two diastereomers, 348 mg, 1.57 mmol) was analogously epoxidized with MCPBA (374 mg) in the presence of NaOAc (143 mg) in dicholomethane (10 ml) to provide *trans*-(7*R*)-10,11-epoxy-1-bisabolen-3-ols (four stereoisomers, 204 mg, 55 %) Rf 0.28 (hexane/ethyl acetate, 4:3). GC/EI-MS (*m/z*, relative abundance): 220 (2, M^+^-18), 165 (28), 138 (16), 134 (48), 132 (61), 119 (52), 109 (40), 105 (34), 93 (72), 91 (50), 79 (33), 77 (35), 71 (54), 69 (25), 67 (25), 59 (27), 55 (32), 43 (100), 41 (43). The data are in agreement with those previously published for RSRS found in *H. halys* extract (Khrimian et al. 2014a). This mixture was tested in the field as lure **#**9 (Fig. 1, Table 1).

A mixture of *cis*-(7*R*)-10,11-epoxy-1-bisabolen-3-ols and *trans*-(7*R*)-10,11-epoxy-1-bisabolen-3-ols, in a 3:1 ratio, was tested as lure **#**6 (Table 1). A crude mixture from the reaction of (7*R*)-4-(6-methylhept-5-en-2-yl) cyclohex-2-enone with methyl lithium (3.30 g) was epoxidized with MCPBA (3.44 g), as described above, and the reaction monitored by TLC. The excess of MCPBA was removed with 10 % Na_2_SO_3_ using Quantofix (Macherey-Nagel, Duren, Germany) peroxide indicator before NaHCO_3_ treatment. The organic extract was thoroughly washed with water, brine, then dried with Na_2_SO_3_ and concentrated to yield a crude mixture (3.10 g) containing 33 % *cis*-10,11-epoxy-1-bisabolen-3-ols, 53 % *trans*-10,11-epoxy-1-bisabolen-3-ols, 9 % product, presumably 1,2,10,11-diepoxybisabolan-3-ol (represented by 16 stereoisomers), and 5 % unidentified products. Presumed 1,2,10,11-diepoxybisabolan-3-ols appeared on the GC as three broad peaks that showed similar mass spectra. GC/EI-MS of 1,2,10,11-diepoxybisabolan-3-ols (*m/z*, relative abundance): 175 (2), 165 (3), 163 (3), 151 (5), 147 (6), 138 (19), 125 (31), 109 (51), 95 (53), 81 (49), 71 (44), 69 (25), 55 (30), 43 (100), 41 (40). GC-CIMS (NH_3_, *m/z*): 272 (M^+^+18), 254 (M^+^), 237, 221, 219, 203. This crude mixture was tested in field trials as lure **#**11 (Table 1).

A part of this crude mixture (2.30 g) was purified by flash chromatography (hexane:ethyl acetate, 5:4) to give three fractions: No.1, 333 mg *cis*-(7*R*)-10,11-epoxy-1-bisabolen-3-ols of 91 % purity; No.2, 230 mg containing 32 % *cis*-(7*R*)-10,11-epoxy-1-bisabolen-3-ols, 65 % *trans*-(7*R*)-10,11-epoxy-1-bisabolen-3-ols, and 3 % unknowns; No.3, 600 mg containing *trans*-(7*R*)-10,11-epoxy-1-bisabolen-3-ols. By mixing No.1 with 126 mg of No.2, we obtained 459 mg of a mixture of *cis*- and *trans*-(7*R*)-10,11-epoxy-1-bisabolen-3-ols in a 3:1 ratio. This product was tested in the field as lure **#**12 (Table 1). Syntheses of chemical lures based on (*S*)- and racemic citronellals are described in Supplementary Materials. The synthetic procedures described above were scaled up to quantities needed for conducting multiple-year field trapping studies.

### Field Bioassays

Pyramid traps of dimensions previously employed for native stink bugs (Hogmire and Leskey 2006; Leskey and Hogmire 2005) and for brown marmorated stink bugs (Leskey et al. 2012d) were used for all trials. Panels were constructed from Sintra® (partially extruded PVC) sheets (Laird Plastics, Pittsburgh, PA, USA) or plywood, and painted with flat black latex exterior paint, based on previous results indicating that adult and nymphal *H. halys* responded in greater numbers to this particular visual stimulus compared with other visual stimuli (Leskey et al. 2012d). Each panel was 1.22 m high, 52 cm wide at the base, and 7 cm wide at the top. Collection jars were constructed as per published dimensions (Hogmire and Leskey 2006) with an internal cone opening of 1.6 cm and trimmed wire edging to reduce escape. In addition, a Hercon Vaportape™ II (Hercon Environmental, Emigsville, PA, USA) was added as a killing agent to prevent escape of insects from traps. Traps were deployed ~5 m from the border of apple and pear orchards at the Appalachian Fruit Research Station or in the border area between wood lots and row crops in Kearneysville, WV, USA, Shepherdstown, WV, USA, Keedysville, MD, USA, and at the Beltsville Agricultural Research Center, MD, USA. Traps were spaced ~50 m apart and were baited with candidate compounds synthesized and impregnated into gray rubber septa or left unbaited as controls. In all trials, an unbaited trap was included and served as a control. Lures were changed every 2 weeks during a trial, as previous work showed that attraction to a septum decreases significantly after this point (D.W. unpublished data). Traps were checked once or twice per week during each trial, with all adults and nymphs counted and removed, and treatment positions rotated by weekly or twice weekly re-randomization. Trial location, timing and composition details are in Table 2.

**Table 2:** Capture rates of *H. halys* relative to the sum of individuals caught in the unbaited control at Appalachian Fruit Research Station, WV, and Beltsville, MD during 2011

		Factor greater than capture by control			
		Adults		Nymphs	
Trial	Treatment	AFRS^a^	Beltsville^b^	AFRS	Beltsville
One 8 Jul—2 Aug	#2	4.3	2.0	2.0	1.9
	#3	5.9	2.8	1.6	2.3
	#4	2.4	1.4	1.3	1.8
Two 8 Aug—19 Aug	#3 (50 mg)	3.9	–	1.9	–
	MDT	2.6	–	2.7	–
Three 11 Aug—6 Sep	#1	1.2	1.1	1.5	0.6
	#2	5.2	2.7	2.7	1.0
	#3	5.6	4.3	2.4	1.1
	#5	6.0	3.7	2.5	1.3
Four 6 Sep—30 Sep	#6	15.3	18.7	1.2	2.6
	#2	3.2	3.3	0.9	1.6
	#3	4.1	2.3	1.3	1.3
	#5	3.5	2.8	1.1	1.9
Five 19 Sep—4 Oct	#6	7.4	–	3.6	–
	#7	1.8	–	1.0	–
	#3	2.3	–	0.8	–
	MDT	5.3	–	6.2	–
Six 30 Sep—20 Oct	#6	7.3	9.6	4.0	1.0
	#8	8.6	15.8	5.0	1.0
	#9	1.1	1.8	1.0	1.0
	#10	20.3	44.0	5.0	0.0

^a^Appalachian Fruit Research Station, Kearneysville, WV

^b^Beltsville Agricultural Research Center, Beltsville, MD

See Table 1 for identities of compounds. MDT = methyl (2*E*, 4*E*, 6*Z*)-decatrieonate

Dose-dependent trials also were conducted with several of the lures, and these included an unbaited control trap in each set of replicates. Dose-dependent trials were conducted with non-pheromonal stereoisomer treatment #3 at 2, 8, and 50 mg during the mid- and late-season from 29 July - 10 August and 12–16 September 2011, respectively, in Kearneysville, WV. Dose-dependent pheromone trials of stereoisomer treatment #6 were conducted at three locations: Shepherdstown, WV (17 June - 12 July), Keedysville, MD (17 June - 12 July), and Beltsville, MD (5 June - 3 July) in 2012; loading rates included 0.1, 1, 10, and 100 mg. In 2013, #6 was combined with 66 mg of methyl (2*E*, 4*E*, 6*Z*)-decatrienoate and tested at 10, 100, and 1000 mg of pheromone in soybean fields from 9–30 Aug 2013 in Jefferson County, WV. To achieve these higher doses, 10 of the 10 mg septa were combined by threading them with wire for the 100 mg treatment, and 100 of the 10 mg septa were combined for the 1000 mg #6 aggregation pheromone treatment. A range of doses above and below 200 mg was used, as this was the dose of pheromone used to trap native stink bugs in prior trapping studies (Krupke et al. 2001). *Halyomorpha halys* adults and nymphs were sampled only visually until 16 August, then with black pyramid traps (adults and nymphs), as described above, on a weekly basis.

### Statistical Analysis

Each field trial in which lures differed qualitatively from each other during a time period, and differed in site location was analyzed with an individual linear mixed model, because each trial contained different treatments, numbers of replicates, and the abundance of *H. halys* was often different among locations. For every model, *H. halys* adults (or nymphs, in some cases) were used as the response variable, and the different lures (or doses of the same lure) were used as the categorical explanatory variable. Nymphs and adults were pooled as a composite response variable from the visual sampling in soybean. The different replications were used as a random blocking factor. Because none of the response variables were normally distributed, the data were log-transformed to conform to the assumptions for an ANOVA. Residuals were inspected to ensure a mean variation around zero with homogenous variances. Pairwise comparisons between lures were conducted within a trial using Tukey’s HSD.

The dose-response trials for pheromonal and non-pheromonal stimuli were analyzed with linear regression, except for the soybean trials with #6 + methyl (2*E*, 4*E*, 6*Z*)-decatrienoate, which were analyzed as above. The capture of *H. halys* in traps was regressed against the concentration of chemical stimulus, with the latter as the independent variable. Because the data did not conform to a normal distribution, they were log-transformed prior to analysis. Regressions were performed separately across sites due to varying absolute numbers of *H. halys*, and adjusted *R* ^2^-values and *P*-values were calculated for each regression. All statistical analyses were carried out in the program JMP Genomics v. 5.0 (SAS Institute, Inc. 2010), and we specified α = 0.05 for all tests.

## Results

### Response to Pheromonal and Non-pheromonal Stereoisomers

**Fig. 2:**
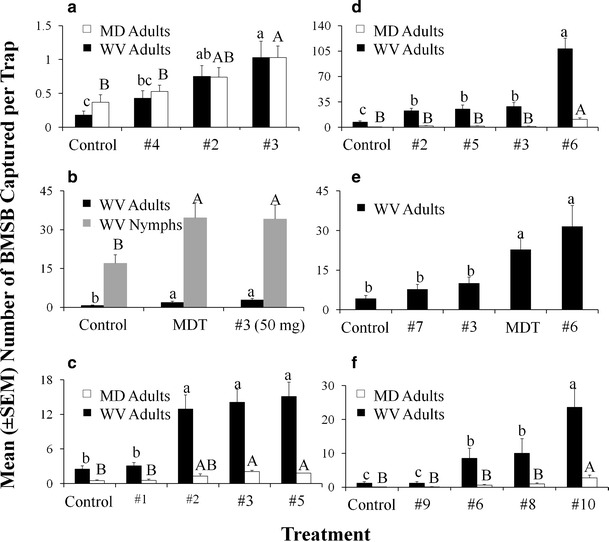
Mean captures (± SE) of *Halyomorpha halys* (BMSB) in black pyramid traps baited with different mixtures of stereoisomers in different trials in 2011 from: **a** 8 July to 2 August (Trial 1), **b** 8 August to 19 August (Trial 2), **c** 11 August to 6 September (Trial 3), **d** 6 September to 30 September (Trial 4), **e** 19 September to 4 October (Trial 5), and **f** 30 September to 20 October (Trial 6). The figure is based on adults captured from Beltsville, MD (*white bars*) or the Appalachian Fruit Research Station, WV (*black bars*), or nymphal data (*grey bars*) from WV. Bars with shared letters are not different from one another, with lower case and upper case letters representing comparisons within location of capture or stage of *H. halys* (Tukey’s HSD, α = 0.05). See Table 1 for identities of compounds. MDT = methyl (2*E*, 4*E*, 6*Z*)-decatrienoate

Lures #2 and #3 contained non-pheromonal stereoisomers; i.e., isomers not produced by *H. halys* (Table 1). Nevertheless, in Trial 1, traps baited with #3 attracted 5.9 and 2.8 times more adults than unbaited traps (control), and yielded different captures in field trials conducted in WV (*F* = 5.97; *df* = 3, 152; *P* < 0.001; Fig. 2a; Table 2) and MD (*F* = 33.7; *df* = 3, 387; *P* <0.001), respectively. Captures in traps baited with #2 were also greater than in unbaited traps in WV. In Trial 2, captures in traps baited with #3 and with methyl (2*E*, 4*E*, 6*Z*)-decatrienoate were greater than in the control for adults (*F* = 3.00; *df* = 2, 211; *P* < 0.05) and nymphs (*F* = 24.8; *df* = 2, 211; *P* < 0.001; Fig. 2b). In Trial 3, traps containing #5, #3 and #2 captured more adults than the control in WV (*F* = 26.0; *df* =4, 191; *P* < 0.001), and those baited with #3 and #5 caught more adults than the control in MD (*F* = 9.57; *df* = 4, 360.9; *P* < 0.001; Fig. 2c). Traps baited with these lures captured 2.7–6.0 times more adults than unbaited traps (Table 2).

In Trial 4, lures #5, #3, and #2 caught greater numbers of *H. halys* adults in WV compared with the control. However, adult captures in traps baited with #6, a treatment containing both stereoisomers of the *H. halys* pheromone, were greater than all other treatments in WV (*F* = 30.2; *df* = 4, 166; *P* < 0.001; Fig. 2d, black bars) and MD (*F* = 35.0; *P* < 0.001; Fig. 2d, white bars; Table 2). Captures in traps baited with #6 were 15.3 and 18.7 times greater than in unbaited traps in WV and MD, respectively (Table 2).

In Trial 5, traps baited with #6 or methyl (2*E*, 4*E*, 6*Z*)-decatrienoate caught greater numbers of adults compared with traps baited with non-pheromonal stereoisomers #3, #7, or unbaited control traps (*F* = 17.9; *df* = 4, 116; *P* < 0.001; Fig. 2e). In this case, captures in traps baited with 10.7 mg of #6 were similar to those containing over 119 mg of methyl (2*E*, 4*E*, 6*Z*)-decatrienoate. Adult captures in traps baited with #6 or methyl (2*E*, 4*E*, 6*Z*)-decatrienoate were >5 times those in unbaited traps (Table 2). In Trial 6, traps baited with #10 (Table 1) captured more adults in WV (*F* = 85.6; *df* = 4, 141; *P* < 0.001) and MD (*F* = 113.3; *df* = 4, 386; *P* < 0.001; Fig. 2f) than any other treatment. Captures in traps baited with #6 and with #8 (containing only the SSRS component of *H. halys* pheromone) also caught greater numbers of adults in WV compared with the control; however, traps baited with #9 (containing only the RSRS component of the *H. halys* pheromone) did not (Tukey’s HSD).

### Pheromonal Purity

**Fig. 3:**
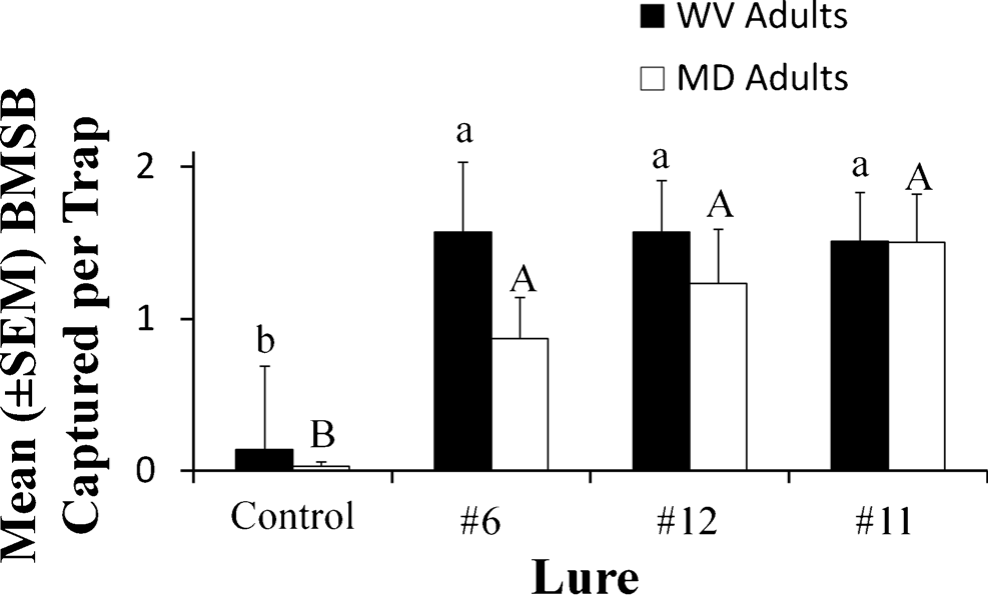
Effect of lures composed of pure stereoisomer or crude mixtures on mean capture (± SE) of *Halyomorpha halys* adults from the Appalachian Fruit Research Station, WV (*black bars*) from 14 May to 7 June or Beltsville, MD (*white bars*) from 8 May to 5 June 2012. Bars with shared letters are not different from one another, with lower and upper case letters representing comparisons within each sampling location (Tukey’s HSD, α = 0.05). See Table 1 for identities of compounds

Regardless of whether lures contained only the two components of the *H. halys* aggregation pheromone (#6), or were composed of pheromonal components and other stereoisomers not produced by *H. halys* (#11 and 12), traps baited with these chemicals captured more adults than unbaited traps and, importantly, had similar catches to one another in WV (*F* = 4.71; *df* = 3, 132; *P* < 0.004) and MD (*F* = 4.56; *df* = 3, 112; *P* < 0.005; Fig. 3). Traps baited with #6, #11, and #12 caught >10 times more individuals than unbaited traps at both locations (Fig. 3).

**Fig. 4:**
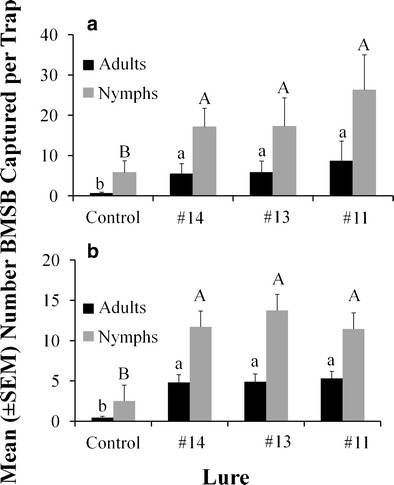
Purity trial showing mean catches of *Halyomorpha halys* (± SE) in **a** West Virginia and **b** Maryland from 12 Aug—4 Sep 2013 for lures that were synthesized using racemic citronellal and were minimally purified by one or no chromatographic separations. Bars with shared letters are not different from one another, with capitalized and lower case letters representing comparisons within each life stage (Tukey’s HSD, α = 0.05). See Table 1 for identities of compounds

Likewise, treatments using inexpensive racemic mixtures of citronellal as the starting material (lures #11, #13, #14) resulted in greater captures of *H. halys* adults and nymphs regardless of whether the sampling site was in WV (Adults: *F* = 24.0; *df* = 3, 72; *P* < 0.001; Nymphs: *F* = 9.98; *df* = 3, 72; *P* < 0.001; Fig. 4a), or MD (Adults: *F* = 4.0; *df* = 3, 72; *P* < 0.01; Nymphs: *F* = 15.7; *df* = 3, 72; *P* < 0.001; Fig. 4b). On average, in WV, adult and nymphal captures in baited traps were almost 11 and 4 times greater than the unbaited control, respectively. Similarly, in MD, the baited traps caught >11 times and almost >5 times the numbers of adults and nymphs, respectively, when compared with the control. Importantly, regardless of whether the baited trap had a lure with *cis*- or *trans*-configurations, or crude mixtures with all 16 stereoisomers and one or no chromatographic purification, *H. halys* trap catch was not affected (Fig. 4 a, b, Tukey’s HSD).

### Dose-Dependent Trials

**Fig. 5:**
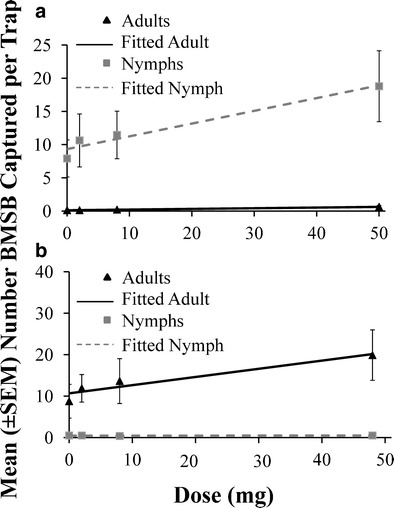
Dose-dependent responses for mean captures (± SE) of *Halyomorpha halys* adults (*black bars*) and nymphs (*grey bars*) in 2011 to different levels of a non-host produced stereoisomer #3 in **a** mid-season (29 Jul—10 Aug), or **b** late season (12–16 Sep) in field trials with black pyramid traps at the Appalachian Fruit Research Station, WV. There was a positive relationship for both nymphs and adults in the mid-season (*nymphs*: Adj *R* ^2^ = 0.94; *P* < 0.001; *y* = 0.192x + 9.31; *adults*: Adj *R* ^2^ = 0.98; *P* < 0.002; *y* = 0.192x + 9.31) and adults in the late season (Adj. *R* ^2^ = 0.88; *P* < 0.05; *y* = 0.197x + 10.7), but not nymphs in the late season (Adj. *R* ^2^ = 0.03; *P* = 68; *y* = 0.0005x + 0.461). See Table 1 for identities of compounds

For #3, a stereoisomer not produced by *H. halys*, a dose-dependent response for nymphs (Adjusted *R* ^2^ = 0.94, *P* < 0.001) and adults (Adj. *R* ^2^ = 0.98, *P* < 0.002; Fig. 5a) was observed in traps baited with 2, 8, and 50 mg compared with unbaited traps in a trial conducted during the mid-season. However, in the late season, although adults continued to exhibit a dose-dependent response to lure #3 (Adj. *R* ^2^ = 0.88, *P* < 0.05; Fig. 5b), very few nymphs were present in the field, and a dose-dependent response was not observed (Adj. *R* ^2^ = 0.03, *P* = 0.68).

**Fig. 6:**
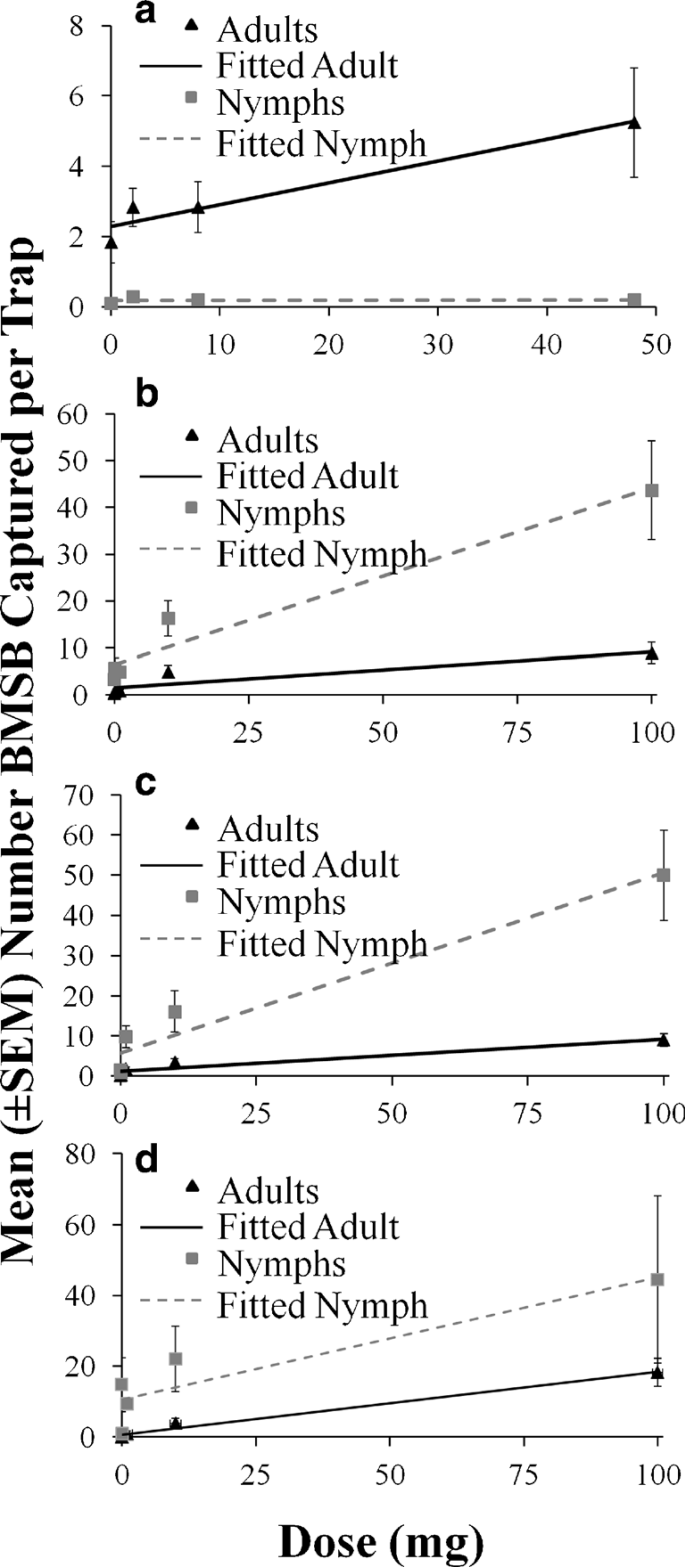
Dose-dependent response for mean captures (± SE) of *Halyomorpha halys* adults (*black bars*) and nymphs (*grey bars*) in West Virginia in **a** late season (7–20 Oct) of 2011 at AFRS, and in mid-season (21 Jun – 9 Aug) of 2012 in **b** Shepherdstown, WV, **c** Keedysville, MD, and **d** Beltsville to different levels of the host-produced lure #6 alone in field trials with black pyramid traps. There was a significant positive relationship between the dose of #6 alone and the BMSB capture for adults at AFRS (*R* ^2^ = 0.92; *P* < 0.001; *y* = 0.062x + 2.29), Shepherdstown (*R* ^2^ = 0.83; *P* < 0.001; y = 0.77x + 1.50), Keedysville (*R* ^2^ = 0.90; *P* < 0.001; *y* = 0.080x + 1.21), and Beltsville (*R* ^2^ = 0.99; *P* < 0.001; *y* = 0.178 x + 0.66), and nymphs at Shepherdstown (*R* ^2^ = 0.96; *P* < 0.001; *y* = 0.378x + 6.40) and Keedysville (*R* ^2^ = 0.93; *P* < 0.001; *y* = 0.450x + 5.69), but not AFRS (*R* ^2^ = 0.02; *P* = 0.63; *y* = 0.0005x + 0.183) or Beltsville (*R* ^2^ = 0.84; *P* = 0.12; *y* = 0.347 x + 10.6). See Table 1 for identities of compounds

In the late-season of 2011, traps baited with increasing amounts of #6 (2, 8, and 50 mg), a treatment containing both pheromonal components of *H. halys*, showed a direct relationship in the number of adults captured with increasing doses (Adj. *R* ^2^ = 0.92, *P* < 0.001; Fig. 6a). However, this was not the case for nymphs (Adj *R* ^2^ = 0.02, *P* = 0.63), likely because few nymphs were present in the field in the late-season. In early-mid season 2012, adults in WV (Adj. *R* ^2^ = 0.83, *P* < 0.001; Fig. 6b), Keedysville, MD (Adj. *R* ^2^ = 0.90, *P* < 0.001; Fig. 6c), and Beltsville, MD (Adj. *R* ^2^ = 0.99, *P* < 0.001; Fig. 6d) continued to show a dose-dependent response to lure #6. Likewise, when there were more nymphs in the field, greater doses of lure #6 resulted in greater captures of nymphs in WV (Adj. *R* ^2^ = 0.96, *P* < 0.001; Fig. 6b) and Keedysville, MD (Adj. *R* ^2^ = 0.93, *P* < 0.001; Fig. 6c), but not in Beltsville (*R* ^2^ = 0.84, *P* = 0.12; Fig. 6d), likely due to the small sample size. The greatest dose of #6 (100 mg) increased the capture of adults by 20–96-fold and nymphs by 13–34-fold, relative to the unbaited control.

**Fig. 7:**
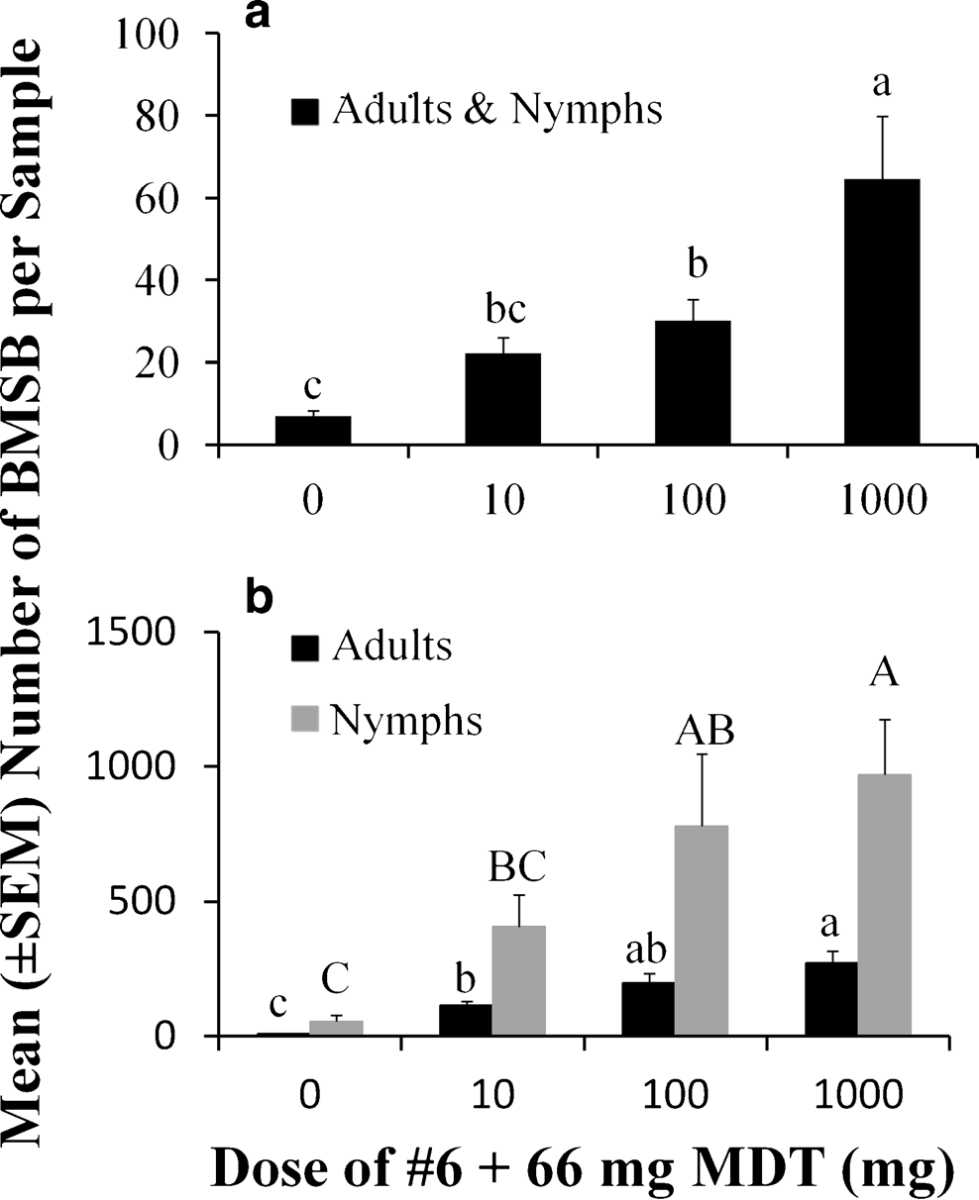
Dose-dependent responses for mean captures (± SE) of *Halyomorpha halys* adults (*black bars*) and nymphs (*grey bars*) to the host-produced compound (Lure #6) and a synergist [methyl (2*E*, 4*E*, 6*Z*)-decatrieonate; MDT] in soybean fields during 2013 evaluated by **a** visual sampling (adults and nymphs pooled; 9–16 Aug) or **b** black pyramid trapping (9–30 Aug). Bars with shared letters are not different from one another, with upper and lower case letters representing comparisons within each life stage of *H. halys* (Tukey’s HSD, α = 0.05). See Table 1 for identities of compounds

In soybeans, when lure #6 + 66 mg of methyl (2*E*, 4*E*, 6*Z*)-decatrienoate was deployed in doses that exceeded the highest dose of previous trials by 10-fold (i.e., 1000 mg), the marked dose-dependent response remained, and resulted in increased captures of adults regardless of sampling method (visual: *F* = 18.6; *df* = 3, 25; *P* < 0.001; Fig. 7a; pyramid trapping: *F* = 3.15; *df* = 3, 57; *P* < 0.04; Fig. 7b). Pyramid trapping also revealed a similar pattern among nymphs in the soybean field for different doses of the same lure (*F* = 4.85; *df* = 3, 57; *P* < 0.005; Fig. 7b). Adults were 9–34 times more abundant in the 1000 mg samples compared to the control, while the nymphal captures were about 18 times greater.

## Discussion

Khrimian et al. (2014a) reported that *H. halys* responded to traps baited with lures comprised of *H. halys* pheromone in the naturally occurring ratio [3.5:1 of (3*S*,6*S*,7*R*,10*S*)-10,11-epoxy-1-bisabolen-3-ol:(3*R*,6*S*,7*R*,10*S*)-10,11-epoxy-1-bisabolen-3-ol] in greater numbers than to either isomer alone, with traps baited with the major SSRS component capturing more insects than those with the minor RSRS component. Similarly, when we compared trap captures with mixed isomer lures containing both components (#6) or those containing the main SSRS (#8) or minor RSRS (#9) components, captures were greatest with both components present, with captures greater in traps with lures containing the main (#8) compared with the minor (#9) component in WV.

Thus, it appears that the presence of non-pheromonal isomers of 10,11-epoxy-1-bisabolen-3-ol were not antagonistic or inhibitory. The pheromone of *Euschistus heros* (F.) was identified as comprising three components, including methyl 2,6,10-trimethyldecanoate, the main component attracting females (Borges et al. 2011). There are eight stereoisomers of methyl 2,6,10-trimethyldecanoate, and bioassays revealed that the SRS isomer was the most attractive and that RSS alone appeared to be repellent. However, when presented as a racemic mixture, there appeared to be no antagonistic or inhibitory effect on overall attractiveness (Borges et al. 1998, 2011). While no repellent isomers were identified for any of the compounds tested in this study, we likewise observed that racemic mixtures of 10,11-epoxy-1-bisabolen-3-ol were broadly attractive to both *H. halys* adults and nymphs.

The lack of a behavioral response by *H. halys* to unnatural isomers is in contrast to other taxa, particularly Lepidoptera, where specific stereoisomers produced by one species can have a potent antagonistic effect on another (Leal et al. 2005; Zhang et al. 2005), thereby requiring high degrees of purity in pheromone formulations used for monitoring (Leskey et al. 2006). Fortunately, this does not appear to be the case with *H. halys*. Indeed, when lures formulated with crude mixtures containing the two pheromone components in equal amounts and a range of other (7*R*)*-*10,11-epoxy-1-bisabolen-3-ols were compared against lures with purified formulations, catches among the mixtures were similar, but greater than in unbaited controls. Indeed, traps baited with mixtures containing all 16 stereoisomers of 10,11-epoxy-1-bisabolen-3-ol (#13, #14, produced from racemic citronellal) were also attractive to *H. halys*. Thus, our work has important practical implications for the commercial production of *H. halys* pheromone. It should be possible to start the synthesis with relatively cheap racemic citronellal instead of (*R*)-citronellal. As a consequence, it may be possible to avoid a costly synthesis while retaining attractive potency. Certainly, these findings will enable companies to formulate pheromone-based products more easily for monitoring and management of *H. halys*.

Interestingly, some (*S*)-citronellal-derived isomers of 10,11-epoxy-1-bisabolen-3-ol not produced by *H. halys* were attractive, signifying that some stereoisomers of (7*S*)-10,11-epoxy-1-bisabolen-3-ol are sufficiently similar to the true pheromone to trigger appropriate behavioral responses. In particular, captures in traps baited with #3 (comprised of four *cis*-and four *trans*-epoxybisabolenols) were greater than unbaited traps in nearly all trials conducted throughout the season. To a lesser extent, traps with #2 were consistently more attractive to *H. halys* adults in WV, although this was not the case at MD field sites, likely due to the lower overall abundance of adults. In contrast, the harlequin bug, *Murgantia histrionica* (Hahn)*,* a species with a male-produced pheromone comprising a 1.4:1 ratio of SSRS and SSRR stereoisomers of 10,11-epoxy-1-bisabolen-3-ol (Khrimian et al. 2014b), was not attracted to other non-pheromonal 10,11-epoxy-1-bisabolen-3-ol stereoisomers tested (Weber et al. 2014b). Attraction of insects to stereoisomers of the pheromone is rare. The sex pheromone of the pink hibiscus mealybug, *MAconellicoccus hirsutus,* comprises a 1:5 mixture of (*R*)-lavandulyl-(*S*)-2-methylbutanoate and (*R*)-maconellyl-(*S*)-2-methylbutanoate. However, it has been demonstrated (Zhang and Amalin 2005; Zhang et al. 2006) that a mixture of the two components with (*S,S*)- configurations also was somewhat active. Thus, the (*S*)-configuration of the acid moiety elicits attraction and the (*R*)-configuration induces inhibition, although there was some degree of tolerance toward chirality in the alcohol portion (Zhang et al. 2006). Further research will focus on finding which stereoisomer(s) of 10,11-epoxy-1-bisabolen-3-ol with 7*S* configuration are attractive to *H. halys*.

Cross-attraction of *H. halys* to methyl (2*E*, 4*E*, 6*Z*)-decatrienoate, the pheromone of *Plautia stali* has been well documented (Aldrich et al. 2007; Khrimian et al. 2008). *Chinavia hilaris* Say [formerly *Acrosternum hilare* (Say)]*,* the green stink bug, is also cross-attracted to this compound (Aldrich et al. 2009). The purpose of cross-attraction in pheromones may include aiding the receiver to locate host plants, dispersing to overwintering sites, and/or benefiting from density-dependent protection from natural enemies (for a more extensive discussion, see Weber et al. 2014a). However, for *H. halys*, there are limitations in terms of the usefulness of this compound, as adults are only attracted to it late in the season, while nymphs are attracted all season (Leskey et al. 2012d). With this caveat, methyl (2*E*, 4*E*, 6*Z*)-decatrienoate has proven to be a powerful synergist for the two-component *H. halys* aggregation pheromone throughout the season (Weber et al. 2014a). Even in the limited trials conducted here, the effect of combining the pheromone and methyl (2*E*, 4*E*, 6*Z*)-decatrienoate resulted in adult captures that were at least 20 and 2–4 times greater than captures in unbaited traps and in traps baited with the pheromone alone, respectively. Thus, using the two-component aggregation pheromone in combination with methyl (2*E*, 4*E*, 6*Z*)-decatrienoate is one way to increase attractiveness and sensitivity of lures for monitoring *H. halys* populations.

A second method to enhance captures in traps is by increasing the amount of material or release rate of the lures. In trials conducted here, we observed increased responses to increasing amounts of the pheromone alone or in combination with methyl (2*E*, 4*E*, 6*Z*)-decatrienoate (Figs. 6 and 7). In previous experiments, a strong response to increasing amounts of methyl (2*E*, 4*E*, 6*Z*)-decatrienoate formulated into lures also was observed (Leskey et al. 2012d). In our study, we also reported increased dose-responses to #3, a chemical not produced by *H. halys*. Thus, there is potential for other non-pheromonal compounds to enhance monitoring and/or behavioral manipulation and management of *H. halys*.

Significant advances have been made toward effective pheromone-based monitoring of *H. halys*. These include the identification of the pheromone (Khrimian et al. 2014a) and methyl (2*E*, 4*E*, 6*Z*)-decatrienoate, a powerful synergist (Weber et al. 2014a). Additionally, black pyramid traps provide a good visual stimulus and collection jars provisioned with a killing agent retain greater numbers of individuals (Leskey et al. 2012c). Previous research has shown that a combination of methyl (2*E*, 4*E*, 6*Z*)-decatrienoate and *H. halys* aggregation pheromone is able to detect *H. halys* populations reliably in both low and high density situations across the United States throughout the active growing season (Leskey et al. 2015). Overall, our work builds on the state-of-the-art by contributing detailed information about the range of pheromonal and non-pheromonal cues to which *H. halys* responds, the purity required for significant behavioral responses, and documentation of dose-dependent responses to pheromonal and non-pheromonal stimuli. We hope this information will help lead to: 1) improved routes for synthesizing pheromones in a more cost-effective manner; 2) future research into identifying other non-pheromonal synergists to attract *H. halys*; and 3) investigation of attract-and-kill approaches for controlling *H. halys* populations while simultaneously reducing insecticide inputs.

## Electronic supplementary material

Below is the link to the electronic supplementary material.

ESM 1 (DOCX 15 kb)
