# Supplementary material for: Behavioral Responses of the Invasive Halyomorpha halys (Stål) to Traps Baited with Stereoisomeric Mixtures of 10,11-Epoxy-1-bisabolen-3-OL
Source: J Chem Ecol. 2015 Apr 9;41(4):418–29. doi: 10.1007/s10886-015-0566-x (PMC4427635; doi:10.1007/s10886-015-0566-x)
Supplement: Supplementary file 1 — (DOCX 15 kb) [file 10886_2015_566_MOESM1_ESM.docx]

BEHAVIORAL RESPONSES OF THE INVASIVE *Halyomorpha halys* (Stål) TO TRAPS BAITED WITH STEREOISOMERIC MIXTURES OF 10,11-EPOXY-1-BISABOLEN-3-OL

TRACY C. LESKEY ^*1^, ASHOT KHRIMIAN^2^, DONALD C. WEBER^2^, JEFFREY C. ALDRICH^2^, BRENT D. SHORT^1^, DOO-HYUNG LEE^3^, WILLIAM R. MORRISON III^1^

*^1^USDA-ARS, Appalachian Fruit Research Station, Kearneysville, WV 25430*

*^2^USDA-ARS Invasive Insect Biocontrol & Behavior Laboratory, Beltsville, MD*

*^3^Department of Life Science, Gachon University, South Korea*

* Corresponding Author: [tracy.leskey@ars.usda.gov](mailto:tracy.leskey@ars.usda.gov)

Supplementary Material

*Syntheses of cis- and trans-(7S)-10,11-epoxy-1-bisabolen-3-ols.*  (*S*)-Citronellal was converted to (7*S*)-4-(6-methylhept-5-en-2-yl)cyclohex-2-enone following Hagiwara et al. 2002. Analogously to the procedure described above, (7*S*)-4-(6-methylhept-5-en-2-yl)cyclohex-2-enone (13.52 g, 65.52 mmol) was dissolved in 800 mL dry ether and allowed to react with methyl lithium (53 ml of 1.6 M in ether; 84.80 mmol) at -15° and -20°C. After regular work-up and flash chromatography on silica gel using hexane : ethyl acetate, 8:1 to 5:1, *cis*-(7*S*)-1,10-bisaboladien-3-ols (4.14 g, 28%), Rf 0.25, hexane : ethyl acetate, 5:1, and *trans*-(7*S*)-1,10-bisaboladien-3-ols (4.75 g, 33%), Rf = 0.17, hexane : ethyl acetate, 5:1, were separated. *cis*-(7*S*)-1,10-Bisaboladien-3-ols were well separated from *trans*-(7*S*)-1,10-bisaboladien-3-ols on HP-5MS capillary column. However, two *cis*-bisaboladienols provided no separation on HP-5MS and two *trans* diastereomers were only partially separated from each other. Relative configurations were assigned following Khrimian et al. 2014a. Mass spectra of *cis*- and *trans*-(7*S*)-1,10-bisaboladien-3-ols matched those previously described in literature (Zahn et al. 2008).

A diastereomeric mixture of *cis*-(7*S*)-1,10-bisaboladien-3-ols (1.157 g, 5.21 mmol) was added to the stirred suspension of dry sodium acetate (471 mg, 5.74 mmol) in dry DCM (35 ml), and the flask was cooled to 0^o^C. MCPBA (1.237 g) was added in small portions at 0^o^C and the stirring was continued at that temperature for 4.5 hours. The mixture was poured into ice-water (20 ml) and extracted with DCM (3 x 20 ml). The combined organic extract was washed with a sodium bicarbonate solution and dried with sodium sulfate. After evaporation of the solvent, the remainder was flash-chromatographed on SiO_2_ with hexane/ethyl acetate, 2:1, to provide *cis-*(7*S*)-10,11-epoxy-1-bisabolen-3-ols (994 mg, 80%) as a mixture of four stereoisomers. This mixture was tested in field as lure **#**2 (Table 1).

GC-EIMS of **#**2 lure (*m/z*, relative abundance): 220 (3, M^+^-18), 205 (3), 202 (3), 187 (4), 165 (10), 159 (9), 145 (10), 147 (14), 138 (13), 134 (38), 132 (42), 121 (35), 119 (52), 109 (27), 105 (35), 93 (87), 91 (66), 79 (42), 77 (44), 71 (53), 69 (25), 67 (22), 59 (26), 55 (33), 43 (100), 41 (48). The data are in agreement with those previously published (Zahn et al. 2008) and matched those obtained for (3*S*,6*S*,7*R*,10*S*)-10,11-epoxy-1-bisabolen-3-ol found in *H. halys* male extract (Khrimian et al. 2014). GC-CIMS (MH_3_, *m/z*, relative abundance): 256 (M^+^+18, 1), 238 (M^+^, 13), 221 (M^+^+1-18,100), 203 (41), 163 (18), 127 (26).

A diastereomeric mixture of *trans*-(7*S*)-1,10-bisaboladien-3-ols (815 mg, 3.67 mmol) was epoxidized with *m*-chloroperbenzoic acid (871 mg) in the presence of sodium acetate (331 mg, 4.04 mmol) in DCM (25 ml) analogously to described above to yield after flash chromatography *trans-*(7*S*)-10,11-epoxy-1-bisabolen-3-ols (682 mg, 78%) as a mixture of four stereoisomers. GC-EIMS (*m/z*, relative abundance): 220 (3, M^+^-18), 205 (3), 202 (3), 187 (4), 165 (14), 159 (10), 145 (14), 147 (13), 138 (9), 134 (40), 132 (58), 121 (36), 119 (61), 109 (28), 105 (40), 93 (93), 91 (68), 79 (35), 77 (46), 71 (51), 69 (24), 67 (22), 59 (25), 55 (33), 43 (100), 41 (49). GC-CIMS (MH_3_, *m/z*, relative abundance): 238 (M^+^, 16), 221 (M^+^+1-18,100), 203 (42), 163 (16), 127 (24). A 3:1 mixture of *cis-*(7*S*)-10,11-epoxy-1-bisabolen-3-ols and *trans-*(7*S*)-10,11-epoxy-1-bisabolen-3-ols was tested in field as lure **#**3 (Table 1).

*Syntheses of mixture of all 16 stereoisomers of 10,11-epoxy-1-bisabolen-3-ols.* Racemic citronellal was converted to 4-(6-methylhept-5-en-2-yl)cyclohex-2-enone (Hagiwara et al. 2002) and the product was purified by distillation (B.p. 109 ^o^C/0.07 mm Hg), then by flash chromatography with hexanes/ethyl acetate, 6:1. A 96%-pure 4-(6-methylhept-5-en-2-yl)cyclohex-2-enone (1.0 g, 4.48 mmol) reacted with methyl lithium (3.93 ml of 1.6 M; 6.29 mmol) in 30 ml dry ether at -20 to -15 ^o^C as described in experiment with (*R*)- citronellal. The crude product (1.08 g) was epoxidized with MCPBA (1.07 g) in the presence of anhydrous sodium acetate (435 mg) in DCM (70 ml) to afford a mixture (1.10 g) containing 35% *cis*-10,11-epoxy-1-bisabolen-3-ols (eight stereoisomers) and 49% *trans*-10,11-epoxy-1-bisabolen-3-ols (eight stereoisomers). This mixture was tested in the field as lure **#**13 (Table 1).

Lure **#**14 was prepared analogously to #13 with the only exception that starting 4-(6-methylhept-5-en-2-yl)cyclohex-2-enone was purified only by distillation and not chromatography. The final products contained 36% *cis*-10,11-epoxy-1-bisabolen-3-ols (eight stereoisomers) and 49% *trans*-10,11-epoxy-1-bisabolen-3-ols (eight stereoisomers).

*Preparation of bisabolane oxide mixture (Mixture A, part of lure 5).* A mixture of *trans*-(7*S*)-1,10-bisaboladien-3-ols (222 mg, 1 mmol; see preparation of **#2** and **#3** lures above) was treated with phosphorus(V) oxychloride (181 µl, 1.80 mmol) in the presence of dry pyridine (440 µl) in dry DCM at 0 ^o^C. The mixture was allowed to warm to room temperature and stirred for 4 h, then it was poured into cold ammonium chloride solution and extracted with DCM. After drying the DCM extract with sodium sulfate, the residue was chromatographed on SiO_2_ to give a hydrocarbon mixture (74 mg) consisted primarily of sesquiphellandrene and zingiberene. The hydrocarbon mixture from a scaled-up reaction (256 mg, 1.25 mmol) was epoxidized with MCPBA (240 mg, 1.39 mmol) in the presence of dry sodium acetate (115 mg, 1,39 mmol) in DCM (9 ml) to yield 47 mg of a mixture consisted of 82% (7*S*)-10,11-epoxy-1,3(15)-bisaboladiene (= (7*S*)-sesquiphellandrene epoxide), 2% (7*S*)-10,11-epoxy-1,3-bisaboladiene ( = (7*S*)-zingiberene epoxide), and 13% (7*S*)-10,11-epoxy-1,3,5-bisaboltriene (= (7*S*)-ar-curcumene epoxide) . The synthetic procedures described above were scaled up to quantities needed for conducting multiple-year field trappings.
